# Hiperplasia pseudoepiteliomatosa: carcinoma escamocelular versus paracoccidioidomicosis oral, un caso con mirada dermatológica

**DOI:** 10.7705/biomedica.6899

**Published:** 2023-08-31

**Authors:** Lina M. Osorio-Cock, Sandra Catalina Jaramillo-Pulgarín, Alba P. Ferrín-Bastidas, Diana Y. Molina-Colorado, Óscar M. Gómez-Guzmán, Alejandra Zuluaga, Juan G.McEwen-Ochoa, Martha E. Urán-Jiménez, María del Pilar Jiménez-Alzate

**Affiliations:** 1 Grupo Micología Médica, Departamento de Microbiología y Parasitología, Facultad de Medicina, Universidad de Antioquia, Medellín, Colombia Universidad de Antioquia Departamento de Microbiología y Parasitología, Facultad de Medicina Universidad de Antioquia Medellín Colombia; 2 Escuela de Ciencias de la Salud, Universidad Pontificia Bolivariana, Medellín, Colombia. Pontificia Universidad Bolivariana Escuela de Ciencias de la Salud Universidad Pontificia Bolivariana Medellín Colombia; 3 Medicáncer, Medellín, Colombia Medicáncer Medellín Colombia; 4 Departamento de Dermatología, Universidad de Antioquia, Medellín, Colombia. Universidad de Antioquia Departamento de Dermatología Universidad de Antioquia Medellín Colombia; 5 Grupo de Biología Celular y Molecular, Corporación para Investigaciones Biológicas, Medellín, Colombia Corporación para Investiga. Biológicas Corporación para Investigaciones Biológicas Medellín Colombia; 6 Grupo de Micología Médica y Experimental, Corporación para Investigaciones Biológicas, Medellín, Colombia Corporación para Investiga. Biológicas Corporación para Investigaciones Biológicas Medellín Colombia

**Keywords:** *Paracoccidioides,* paracoccidioidomicosis, carcinoma de células escamosas, diagnóstico diferencial, micosis, reacción en cadena en tiempo real de la polimerasa, *Paracoccidioides,* paracoccidioidomycosis, carcinoma, squamous cell, diagnosis, differential, mycoses, real-time polymerase chain reaction

## Abstract

La paracoccidioidomicosis es una micosis sistémica endémica en Latinoamérica. La presentación más frecuente compromete crónicamente los pulmones, la piel y las mucosas. Al inicio, este paciente presentó, por varios años, una lesión única en la mucosa oral que, en ausencia de otros síntomas, se relacionó con una neoplasia maligna, específicamente con un carcinoma escamocelular.

La diferenciación entre los dos diagnósticos se hace mediante un examen directo, un estudio histopatológico y cultivos iniciales y subsecuentes. Sin embargo, tales estudios no fueron concluyentes. Después de varias consultas y pruebas, con los resultados del examen directo, la inmunodifusión y la PCR en tiempo real se confirmó el diagnóstico de paracoccidioidomicosis crónica multifocal.

Este caso alerta sobre la ausencia de sospecha clínica de micosis endémicas, dada la presencia de lesiones mucocutaneas que pueden ser producidas por hongos como *Paracoccidioides* spp, y la importancia de considerarlas entre los diagnósticos diferenciales.

## Abstract (en)

Paracoccidioidomycosis is a systemic mycosis endemic in Latin America. The most frequent form involves a chronic compromise of the lungs, skin, and mucosa. The patient started with a single oral lesion that lasted for several years. The absence of other symptoms pointed out a possible malignant neoplasm, specifically a squamous cell carcinoma.

Differentiation between both diagnoses-fungal infection and carcinoma-depends on the results of the direct examination, the histopathological study, and the initial and subsequent cultures. However, in this case, those findings were not conclusive. The coexistence of both diagnoses is frequent and increases the diagnostic challenge. After several consultations and tests, direct examination, immunodiffusion and real-time PCR findings the multifocal chronic paracoccidioidomycosis diagnosis was confirmed.

This case warns about a systematical absence of clinical suspicion of endemic mycoses before the appereance of mucocutaneous lesions, which can be produced by fungi like *Paracoccidioides* spp, and the importance of considering those mycoses among the differential diagnoses.

La paracoccidioidomicosis es una de las micosis sistémicas y endémicas más frecuentes en Latinoamérica ^1^^,^^2^. Aún no se conoce el hábitat preciso de *Paracoccidioides* spp., pero se sospecha que es el suelo de los entornos rurales. Los principales hospederos accidentales del hongo son el hombre y el armadillo, especialmente la especie *Dasypus novemcínctus,* aunque otros animales también han sido implicados, como aquellos que probablemente se infectan en ambientes rurales o periurbanos ^2^.

La enfermedad es más común en hombres agricultores (relación hombre-mujer de 11:1) ^2^ y se desarrolla principalmente en los pulmones, después de la inhalación de propágulos infectantes como aleurioconididas y fragmentos de hifas ^1^^,^^2^. Si la infección no es contenida, se pueden presentar dos formas clínicas: 1) aguda o subaguda, más común en niños, adolescentes y personas inmunocomprometidas; y 2) crónica, más frecuente en la población general ^1^^,^^2^. La presentación crónica compromete usualmente los pulmones, la piel y las mucosas; ocasionalmente, las glándulas suprarrenales, los ganglios linfáticos y el sistema nervioso, entre otros sitios ^2^. Los pulmones son el sitio primario de la infección que, con frecuencia, se manifiesta con tos y disnea ^1^^,^^2^, aunque los síntomas pueden pasar desapercibidos y los hallazgos de la auscultación pueden ser normales ^3^. La presentación típica en la piel y las mucosas son úlceras dolorosas, con fondo granulomatoso y puntos hemorrágicos en el rostro y la mucosa oral, nasal, faríngea o gastrointestinal ^1^^,^^2^.

La presentación de una lesión oral única en ausencia de otros síntomas hace que el principal diagnóstico diferencial sea una neoplasia maligna, particularmente un carcinoma escamocelular ^4^^-^^6^. En la literatura se han reportado algunos casos similares en los que la diferenciación se ha realizado mediante el examen directo y el estudio histopatológico.

En algunos casos el estudio histopatológico no es concluyente por lo cual se reportan los hallazgos como hiperplasia pseudoepiteliomatosa: una proliferación reactiva, resultado de un mecanismo de eliminación transepitelial del material que es reconocido como extraño por la respuesta inmunológica del hospedero.

La hiperplasia pseudoepiteliomatosa se observa en respuesta a una amplia variedad de condiciones como: infecciones (especialmente crónicas), neoplasias (el carcinoma escamocelular es una de ellas), inflamación y trauma ^7^. No obstante, en este caso el examen directo, el estudio histopatológico y los cultivos iniciales y subsecuentes no fueron concluyentes.

## Presentación de caso

**Figura 1:**
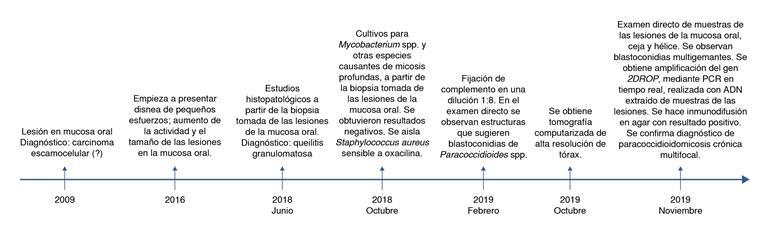
Línea de tiempo del diagnóstico de paracoccidioidomicosís crónica multifocal en el paciente del caso presentado

Se presenta el caso de un hombre de 56 años, residente en el suroeste antioqueño, agricultor, que consultó al Servicio de Dermatología por una lesión dolorosa en la mucosa oral, de nueve años de evolución (figura 1). Refiere como antecedente personal alto consumo de tabaco (30 paquetes al año).

**Figura 2:**
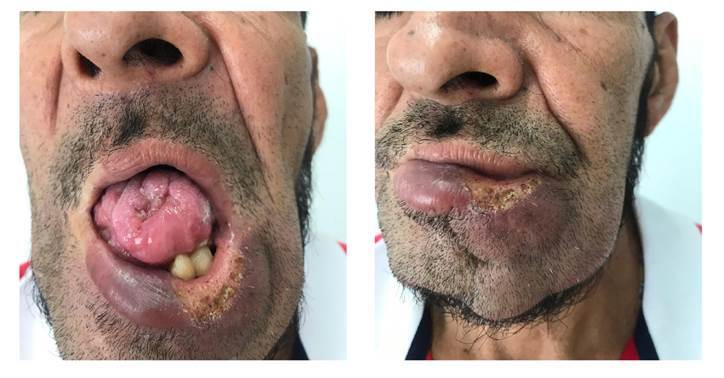
Se aprecia la lesión de la lengua, la mucosa oral y los labios, en forma de placa violácea infiltrada con úlcera central granulomatosa, con compromiso de la mucosa oral y el mentón; cambios fibrocicatriciales en la lengua y las encías, y pérdida de numerosas piezas dentales y cerca de la mitad del cuerpo lingual.

En el examen físico, se observó una placa violácea infiltrada con úlcera central granulomatosa con amplio compromiso de la mucosa oral y el mentón, con presencia de cambios fibrocicatriciales en la lengua y las encías, pérdida de numerosas piezas dentales y cerca de la mitad del cuerpo lingual (figura 2). La impresión diagnóstica inicial fue un carcinoma escamocelular.

El paciente regresó a consulta nueve años más tarde por presentar disnea y aumento del tamaño de las lesiones de la mucosa oral, razón por la cual se obtuvo una biopsia. El estudio histopatológico evidenció un proceso inflamatorio de tipo granulomatoso supurativo. La sospecha diagnóstica cambió a una micosis profunda o una enfermedad por micobacterias. Se solicitaron las tinciones de Ziehl-Neelsen, Ziehl-Neelsen modificado, ácido peryódico de Schiff y metenamina de plata (Grocott-Gomori), para la identificación de microorganismos, pero los resultados fueron negativos.

El paciente volvió a consulta cuatro meses más tarde y se practicaron nuevas biopsias de las lesiones con las cuales se hicieron cultivos para bacterias, *Mycobacterium* spp. y agentes causales de micosis profundas. Los cultivos para *Mycobacterium tuberculosis* y los de hongos de micosis profundas fueron negativos después de ocho semanas de incubación; únicamente se aisló *Staphylococcus aureus,* sensible a oxacilina, para lo cual se le prescribió tñmetopñm-sulfametoxazol por siete días. La conclusión de este primer estudio histopatológico fue queilitis granulomatosa.

**Figura 3:**
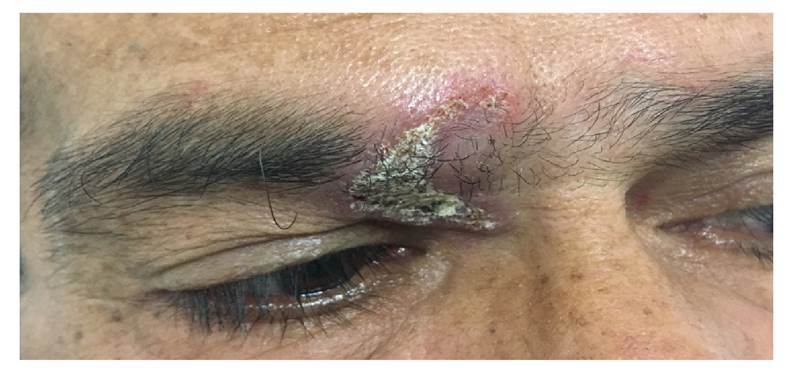
Lesión granulomatosa en la ceja derecha: placa infiltrada cubierta con costra serohemática

El paciente volvió a consultar dos años después ya que su condición clínica progresó con presencia de nueva placa violácea, infiltrada, con costra serohemática, dolorosa en la ceja derecha (figura 3) sumada a las lesiones de la mucosa oral que se describieron anteriormente. Se le realizó un hemograma tipo III (cuadro 1), prueba para el virus de la inmunodeficiencia humana (negativa), serología para la detección de treponema causante de sífilis (no reactiva), creatinina en suero con valor de 1,42 mg/dl y una tomografía de tórax de alta resolución (TACAR).

**Cuadro 1:** Resultados del hemograma tipo III realizado al paciente.

Serie blanca			Serie roja
Leucocitos: 11,4 x 103/μI**		Eritrocitos: 5,74 x 106/μΙ**	
	Linfocitos: 25,4 % (2,9 x 103/μΙ)		Hemoglobina: 16,5 g/dL
	Monocitos: 10,7 % (1,2 x 103/μΙ)**		Hematocrito: 48,8 %
	Granulocitos: 63,9 % (7,3 x 103/μΙ)**		Volumen corpuscular medio: 85,1 fl
**Plaquetas**		Hemoglobina corpuscular media: 28,7 pg	
	Plaquetas: 373 x 103/μΙ	Concentración de la hemoglobina corpuscular media: 33,8 g/dl	
	Volumen plaquetario medio: 7,1 fl**	RDW-CV: 10,5%	
Distribución plaquetaria: 10 fl		RDW-DE: 33,8 fl**	
Plaquetocrito: 0,26 %			

RDW: *red cell distribution width* (amplitud de distribución eritrocitaria): CV: coeficiente de variación; DE: desviación estándar

** parámetros alterados

La sospecha diagnóstica de una micosis profunda persistió, por lo cual el paciente se remitió al Laboratorio de Micología Médica de la Facultad de Medicina de la Universidad de Antioquia, en donde se profundizó en la historia clínica: el paciente refirió disnea de medianos esfuerzos, sudoración nocturna, pérdida de peso y tos. Además, no presentó mejoría de las lesiones a pesar del tratamiento antibiótico. Se tomaron nuevas muestras de las lesiones para estudio. En la microscopía directa se observaron estructuras compatibles con blastoconidias grandes, de contornos irregulares y sin contenido en su interior, sugestivas de *Paracoccidioides* spp. Sin embargo, en el cultivo solo se obtuvo el crecimiento de colonias de *Candida albicans.* Se realizó fijación del complemento para *Paracoccidioides spp.* y se encontró reactividad en una dilución de 1:8 que reforzó el diagnóstico probable de paracoccidioidomicosis.

**Figura 4:**
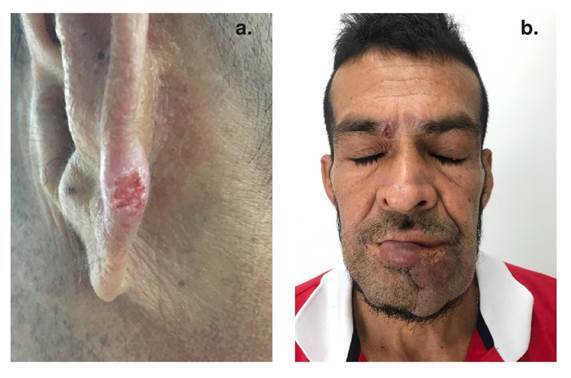
a. Lesión en la hélice izquierda: placa violácea ulcerada. b. Foto panorámica en la que se puede observar el compromiso de las lesiones en labio inferior y ceja derecha.

**Figura 5:**
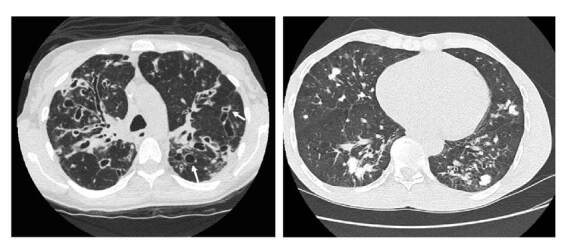
Tomografía computarizada de alta resolución de tórax (TACAR). Las flechas de la imagen izquierda indican múltiples granulomas calcificados distribuidos en todos los segmentos y los lóbulos pulmonares con predominio en las bases y la región parahiliar.

Luego del diagnóstico probable de paracoccidioidomicosis se continuó con la valoración clínica del paciente durante nueve meses, quien asistió al seguimiento con una nueva placa violácea dolorosa en la hélice izquierda (figura 4), aumento de la extensión de la lesión de la mucosa oral y persistencia de la lesión de la ceja izquierda. Además, se encontraron adenopatías cervicales e inguinales y se auscultó hipoventilación en las bases pulmonares con crepitación en la base pulmonar izquierda y sibilandas de predominio superior. En el reporte de latomografía se describieron múltiples granulomas calcificados, distribuidos en todos los segmentos y los lóbulos pulmonares, con predominio en las bases y la región parahiliar, asociados a cambios fibrocicatriciales. También se observaron algunos granulomas calcificados en los lóbulos superiores. Al valorar las lesiones más calcificadas en los lóbulos inferiores, se observó que algunas de ellas contenían material no calcificado en los bronquios dilatados, lo que sugirió la posibilidad de bronquiectasias (figuras 5A y 5B). Se repitió la toma de muestras para estudio con raspado exhaustivo de las lesiones de la mucosa oral, la ceja y la hélice izquierda, y se hizo venopunción para la obtención de suero.

**Figura 6:**
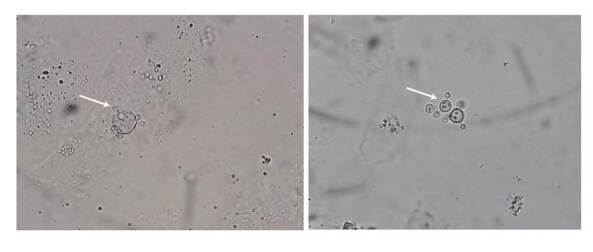
Examen directo de las muestras de raspado de la lesión de la lengua, la mucosa oral, el labio, la ceja y la hélice izquierda, tratadas con solución del 10 % de hidróxido de potasio (KOH) y 0,2 % de azul de Evans. Se observan blastoconidias multigemantes de pared gruesa con inclusiones intracitosplasmáticas.

Se realizó inmunodifusión en gel de doble agar para la detección de anticuerpos contra *Paracoccidioides* spp. con resultado positivo. En el examen directo, con solución del 10 % de hidróxido de potasio, se observaron blastoconidias multigemantes de pared gruesa con inclusiones citoplasmáticas (figura 6). Asimismo, se realizó PCR en tiempo real con SYBR Green y ADN extraído de las muestras tomadas por raspado de las lesiones. La PCR se realizó con iniciadores dirigidos a la amplificación específica de una región genómica única de *Paracoccidioides* spp. denominada *2DROP*^8^. El ADN del hongo se amplificó en la PCR en tiempo real y se verificó con el análisis de la curva de fusión y por electroforesis en gel de agarosa.

Con los resultados tanto del examen directo, la inmunodifusión y la PCR en tiempo real se confirmó el diagnóstico de paracoccidioidomicosis crónica multifocal. Se prescribió tratamiento al paciente de 600 mg de itraconazol por día, durante tres días. Posteriormente se dosificó con 200 mg por día, mínimo por un año, previa verificación del perfil hepático normal. En los seguimientos mensuales que se le realizaron al paciente presentó mejoría clínica satisfactoria.

### Consideraciones éticas

El Comité de Ética de la Facultad de Medicina de la Universidad de Antioquia avaló el consentimiento informado, usado para la evaluación del paciente, la toma y el procesamiento de las muestras; asimismo, el uso de la información del paciente, de las fotografías de las lesiones y la publicación de los resultados fue autorizado por el paciente mediante su firma del consentimiento informado avalado.

## Discusión

Este caso tiene algunas características representativas de la enfermedad. Por ejemplo, sobre la epidemiología de la paracoccidioidomicosis crónica, esta ocurre en su mayoría en personas mayores de 30 años ^2^, predominantemente en hombres, en una proporción de 11:1 ^9^ y afecta principalmente a individuos con labores relacionadas con la tierra, como agricultores ^2^.

En los pacientes, los sitios más frecuentemente involucrados son los pulmones (76,7 %) y las mucosas (63 %) ^10^. Sin embargo, en la auscultación se encontraron mínimas anormalidades en comparación con los hallazgos radiográficos, con una clara disociación entre los síntomas y los estudios radiológicos o de otras imágenes diagnósticas ^2^. La desatención a los escasos síntomas pulmonares por parte del paciente y del personal sanitario, sumado a la presencia de un alto factor de riesgo para enfermedades pulmonares como el alto consumo de tabaco, influyó inicialmente al considerar otras opciones diagnósticas. Más importante, la presencia de una única úlcera en la mucosa oral, en ausencia aparente de otros síntomas, y la exposición prolongada al tabaco, hizo que la primera impresión diagnóstica fuera una neoplasia maligna, correspondiente a un carcinoma escamocelular ^4^^-^^6^.

En la literatura se han documentado casos semejantes. Usualmente el diagnóstico diferencial se logra mediante el examen directo y el estudio histopatológico. El primero con una sensibilidad cercana al 74,5 %, que varía dependiendo de la muestra, y el segundo con una sensibilidad del 96,7 % ^11^. Además, los cultivos son positivos en el 80 % de los casos ^12^. Sin embargo, en este caso el diagnóstico no se logró, al principio, por estos métodos. Si bien se obtuvieron indicios de una infección micótica en la primera oportunidad, en una segunda ocasión se sospechó de *Paraccocidiodes* spp. al observar unas estructuras compatibles con blastoconidias grandes, de contornos irregulares y sin contenido en su interior.

Hallazgos similares se han descrito antes en el trabajo de Restrepo, en el 2000, en el cual se estudiaron aspectos morfológicos de *Paracoccidioides brasiiiensis* en tejido de ganglios linfáticos de pacientes con enfermedad activa y otros con enfermedad latente. En esta última, observaron formas irregulares y atípicas del hongo ^13^^)^. Solo se logró confirmar el diagnóstico en una tercera instancia, en un laboratorio especializado, donde se maximizaron las medidas para obtener una adecuada muestra y en donde se pudo incluir una prueba sensible de biología molecular como la PCR en tiempo real. La observación del hongo en el examen directo y la detección del ADN de *Paracoccidioide*s spp. en la muestra permitieron, de una forma rápida (menos de 24 horas después de tomada la muestra), confirmar la sospecha clínica de paracoccidioidomicosis.-

Es importante tener en cuenta la dificultad del diagnóstico ante la similitud clínica entre la hiperplasia pseudoepiteliomatosa con otras enfermedades granulomatosas o incluso con un carcinoma escamocelular ^4^^-^^6^. Aun así, la displasia y las mitosis atípicas encontradas en el carcinoma escamocelular no se ven en la paracoccidioidomicosis ^4^^-^^6^, pero la coexistencia de ambos diagnósticos en el 58 % de los casos ^14^ impide llegar a un diagnóstico preciso.

## Conclusión

En este caso se resalta la perseverancia requerida para lograr el diagnóstico de las presentaciones clínicas de las micosis sistémicas endémicas, la importancia de indagar por síntomas o signos que el paciente no manifiesta (como los pulmonares), la consideración de los diagnósticos diferenciales y la intensificación de las medidas para mejorar el desempeño diagnóstico de las pruebas. Aun así, los resultados pueden ser inconclusos o incluso fallar si no se trata de laboratorios con experiencia en este tipo de micosis.
